# Brain microvascular calcification is increased in human donors with dementia compared to elderly controls: a pilot study

**DOI:** 10.3389/fnagi.2025.1557625

**Published:** 2025-06-17

**Authors:** Kelly A. Borges, Isabelle Lombardi, Mackenzie Sivilli, Joseph Aabye, Isabella Romano, Saud A. Nasruddin, Mugdha V. Padalkar, Daniel Moussouros, Olga V. Savinova

**Affiliations:** Department of Biomedical Sciences, New York Institute of Technology College of Osteopathic Medicine, Old Westbury, NY, United States

**Keywords:** vascular calcification, vascular dementia, Alzheimer's disease, intracranial calcification, neurovascular, microvascular, cerebral small vessel disease

## Abstract

**Introduction:**

Intracranial vascular calcification has been observed in the setting of both Alzheimer's disease (AD) and vascular dementia. Increased calcification in intracranial and extracranial arteries is associated with an increased risk of dementia; however, less is known about the prevalence and implications of microvascular calcification in AD and related dementias. In this study, we compared microvascular calcification in AD-relevant brain regions between human donors with vs. without dementia and/or late-onset AD diagnoses.

**Methods:**

Brain tissue was sampled bilaterally from basal ganglia, hippocampus, posterior cingulate cortex, substantia nigra, and subventricular zone, along with bilateral carotid arteries in a cohort of human donor cadavers with and without dementia at death (*n* = 23, 61% females, 86.4 ± 7.9 years of age). An additional cohort included postmortem posterior cingulate cortex samples from NIH NeuroBioBank donors with and without confirmed late-onset AD (*n* = 10, 40% females, 78.3 ± 2.1 years of age). All samples were scanned by micro-computed tomography. Vascular calcification was quantified as the sum of voxels at an intensity of ≥130 Hounsfield units in a standardized tissue volume. Findings were confirmed by histology.

**Results:**

Our findings indicate higher odds of dementia per one quartile increase in microvascular calcification volume in the hippocampus [OR 9.601 (CI 2.518, 86.803), *p* = 0.0091], posterior cingulate cortex [OR 2.894 (CI 1.222, 8.923), *p* = 0.0302], and subventricular zone [OR 2.851 (CI 1.153, 9.482), *p* = 0.0427]. Similarly, in posterior cingulate cortex samples from the NeuroBioBank, significantly higher microvascular calcification was observed in late-onset AD cases [median 0.0153 (IQR 0.0075, 0.0581), % by volume] compared to controls [median 0.0024 (IQR 0.0016, 0.0104), % by volume; *p* = 0.0265]. Internal carotid calcification was significantly associated with microvascular calcification in the basal ganglia [OR 1.699 (CI 1.156, 2.496), *p* = 0.0093], hippocampus [OR 1.580 (CI 1.056, 2.366), *p* = 0.0281], and posterior cingulate cortex [OR 1.524 (CI 1.009, 2.299), *p* = 0.0452].

**Discussion:**

Our findings indicate that microvascular calcification impacts brain regions relevant to morphologic changes (hippocampus) and hypoperfusion (posterior cingulate cortex) in AD. Our study expands on a recent report of increased brain calcification in the setting of AD, suggesting that microvascular calcification carries pathophysiological significance in the development and/or progression of AD and related dementias.

## 1 Introduction

Cardiovascular disease (CVD) and Alzheimer's disease (AD) are among the leading causes of death and disability in the United States (Xu et al., 2023), and the incidence of AD is rising disproportionately to the increase in the elderly population (Alzheimer's Association, 2022). At least one-third of AD-related dementias are attributable to modifiable atherosclerotic CVD risk factors, such as hypertension and hyperlipidemia (Saeed et al., 2023; Chen and Moe, 2012; Demer and Tintut, 2008). While growing evidence supports a strong and potentially causal association between CVD and AD, the mechanisms linking their pathophysiology remain in question. In light of the shortcomings of the beta-amyloid (Aβ) hypothesis, vascular contributions to AD etiology are being studied more closely (Eisenmenger et al., 2023).

Vascular calcification (VC) is an age-related phenomenon associated with all-cause mortality and an independent predictor of cardiovascular and cerebrovascular events (Hermann et al., 2013; Detrano et al., 2008). VC results in impaired vascular compliance, abnormal vasomotor response, and reduced tissue perfusion (Wang et al., 2006; Nigwekar et al., 2018). Independent of age, systemic VC can also occur in the setting of atherosclerotic CVD, chronic kidney disease, diabetes, and warfarin use (Johnson et al., 2006; Demer and Tintut, 2019; Towler, 2013; Kosciuszek et al., 2022). The risk of incident dementia is increased in these conditions (Bos et al., 2015; Murray et al., 2021; Wang et al., 2022; Chen et al., 2018). Moreover, larger calcification volume in both intracranial and extracranial arteries has been associated with a higher risk of dementia (van den Beukel et al., 2023; Bos et al., 2015).

The most characteristic morphological finding in AD is reduced hippocampal volume or medial temporal lobe atrophy (Burton et al., 2008). Hippocampal volumetry using age-corrected norms predicts the progression of mild cognitive impairment (MCI) to dementia (Burton et al., 2008; Frisoni et al., 2010; Adak et al., 2004). Magnetic resonance imaging (MRI) studies have demonstrated that blood-brain barrier (BBB) breakdown, evident as cerebral microhemorrhage, precedes hippocampal atrophy in individuals with MCI and AD, serving as an early imaging biomarker of cognitive dysfunction (Montagne et al., 2015; Nation et al., 2019; Thrippleton et al., 2019; Shams et al., 2014; Barisano et al., 2022). Further, ^18^F-fluorodeoxyglucose positron emission tomography (FDG-PET) and single-photon emission computed tomography (SPECT) neuroimaging studies reveal hypometabolism and hypoperfusion throughout the temporal, parietal, and prefrontal cortices in AD (Rabinovici et al., 2011; Small et al., 2000; Valla et al., 2001). FDG-PET studies show that these decrements appear years prior to AD symptoms in carriers of the ε4 allele of the apolipoprotein E gene (*APOE4*). Among brain regions affected in these individuals, the posterior cingulate cortex shows the earliest and largest decrement in metabolism (Small et al., 2000; Valla et al., 2001). The posterior cingulate cortex provides neural input to the hippocampus. Chronic hypoperfusion and hypometabolism of the posterior cingulate cortex, therefore, functionally disconnect it from the hippocampus, resulting in hippocampal atrophy. Neurodegeneration in AD, defined by structural volume loss and/or decreased neuronal metabolism (Jack et al., 2016), thus may be attributable to chronic hypoperfusion. Vascular disease underlying hypoperfusion also contributes to the hypothesis that AD arises as a consequence of mitochondrial dysfunction in these brain regions (Valla et al., 2001). Studies using functional MRI, infrared spectroscopy to measure hemoglobin oxygen saturation, and biochemical analyses of myelin proteins, however, have shown that the reduction in blood flow in AD is primarily due to inadequate blood supply rather than reduced metabolic demand (Love and Miners, 2015).

Chronic hypoperfusion results from compensatory vascular remodeling that occurs with longstanding vascular disease. With age and hypertension, repeated mechanical stress induced by pulsatility induces biomechanical fatigue of vascular wall components in the load-bearing tunica media. Degeneration of elastic fibers progresses with deposition of collagenous material, ground substance, and hydroxyapatite in the vessel wall. VC results in increased vascular stiffness, further increasing systolic pressure (Laurent and Boutouyrie, 2015). Mathematical modeling suggests vessel pulsations provide the force to drive perivascular drainage; thus, vascular stiffness may also reduce the clearance of cerebral Aβ (Vasilevko et al., 2010). Therefore, it is plausible that VC impairs both cerebral blood flow and neurotoxin clearance. Hippocampal VC has been observed in the setting of AD, as noted incidentally on radiologic and histopathologic reports (Fujita et al., 2003; Saade et al., 2019; Maheshwari et al., 2022). Age-related vascular dysfunction may play a role in the development of dementia via blood-brain barrier (BBB) breakdown and hypoperfusion in brain regions associated with AD (Barisano et al., 2022; Nation et al., 2019; Austin et al., 2011).

A study of 2,339 stroke-free and dementia-free elderly participants followed for over a decade demonstrated an association between calcification in the internal carotid and vertebrobasilar arteries and increased dementia risk, mediated partly through cerebral small vessel disease (van den Beukel et al., 2023). Further interrogation of calcification in the brain's microcirculation is warranted to understand its implication in the development of dementia. Clinical imaging modalities, however, are not sensitive to detecting microcalcification in the brain. In this pilot study, we demonstrate that calcification in brain microvasculature can be quantified precisely using high-resolution micro-computed tomography (microCT) *ex vivo* and we utilize this method to quantify vascular calcification in the brains and internal carotid arteries of human donors with and without dementia and late-onset AD. We additionally expand on prior studies by examining brain regions in which differential gene expression has been observed in the setting of AD (Greenwood et al., 2020). Our primary objective was to compare microvascular calcification volume by brain region between elderly donors with vs. without dementia and late-onset AD. We hypothesized that higher volumes of microvascular calcification would be detected in brain regions associated with hypoperfusion in AD in donors with dementia and/or late-onset AD compared to elderly controls. As an exploratory objective, we aimed to examine the relationship between internal carotid artery calcification and brain microvascular calcification by region. We hypothesized that higher calcification volumes in the internal carotid artery would be associated with higher volumes of brain microvascular calcification.

## 2 Materials and methods

### 2.1 Human tissue samples

**Figure 1:**
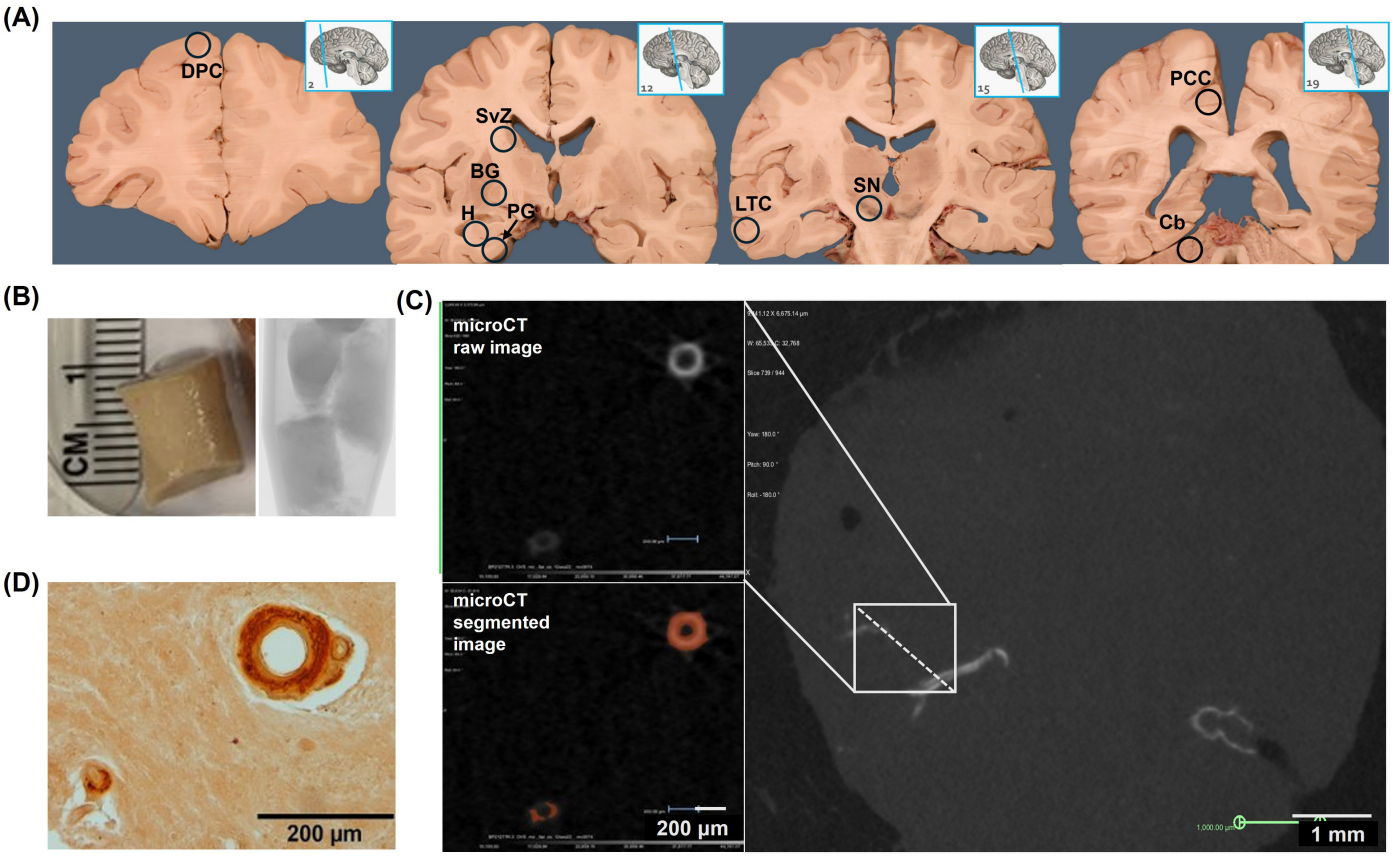
Radiologic and histologic detection of microvascular calcification in human brain tissue (Cohort 1). **(A)** Bilaterally sampled regions included dorsolateral prefrontal cortex (DPC), subventricular zone (SVZ), basal ganglia (BG), hippocampus (H), parahippocampal gyrus (PG), lateral temporal cortex (LTC), substantia nigra (SN), posterior cingulate cortex (PCC), and cerebellum (Cb). **(B)** Tissue biopsies were immersed in mineral oil and scanned by microCT at 10 μm resolution. **(C)** MicroCT images were reconstructed in 3D and segmented at an intensity level equivalent to 130 Hounsfield units (HU). **(D)** Following microCT, tissues were sectioned at 5 μm thickness, stained with alizarin red, and imaged by bright-field microscopy. The images in **(A)** were derived under a Creative Commons license from the website Functional Neuroanatomy, https://neuroanatomy.ca/horizontals.html, associated with the University of British Columbia. We modified the original content by repositioning the sagittal brain images and superimposing circled abbreviations on the coronal brain images. The original images' licensing applies to the altered images.

Cohort 1 brain tissue samples were obtained from donor cadavers at the New York Institute of Technology College of Osteopathic Medicine (NYITCOM) Anatomy Lab in accordance with approval by NYITCOM's Cadaver Use Committee. Brain tissue and extracranial carotid arteries were sampled bilaterally from 23 human donor cadavers at NYITCOM; 11 donors had a diagnosis of dementia documented in their death certificate (three of which were diagnosed with AD specifically), and12 donors did not have documented dementia. Samples were retrieved from cases and controls that were matched for sex and approximate age (Table 1). Carotid arteries were dissected bilaterally by making incisions ~2 cm above and below the carotid bifurcation. Approximately 500 mg of tissue was sampled bilaterally from basal ganglia at the level of the anterior commissure, hippocampus (including dentate gyrus), posterior cingulate cortex [Brodmann area (BA) 23 at the level of the splenium], substantia nigra, and subventricular zone from available cadavers with adequately preserved brain tissue (Figure 1A). Subjects with desiccated/nonviable brain tissue were omitted from biopsy collection; sample sizes for each brain region are noted in Table 2. Hippocampus and posterior cingulate cortex were the primary brain regions of interest in this study for their relevance in AD; prior studies offer evidence of hippocampal tissue calcification (Tsolaki et al., 2022) and posterior cingulate hypoperfusion in the setting of AD (Small et al., 2000; Valla et al., 2001). Basal ganglia are a known site of age-related microvascular calcification in the general elderly population (Bartstra et al., 2020) and served as a positive control for the detection of calcification by microCT; substantia nigra served as a subcortical control site not associated with AD or calcification; subventricular zone served as a subcortical site of adult neurogenesis (Park et al., 2023).

**Table 1:** Characteristics of the study population in Cohort 1 (NYITCOM dementia cohort).

	**Control (*N* = 12)**	**Dementia (*N* = 11)**	***p* (Fisher's exact)**
Sex, *N* female (%)	7 (58%)	7 (64%)	>0.9999
Age, mean years ± SD	85 ± 8	88 ± 8	0.4668^a^
History^b^ of CVD, *N* (%)	6 (50%)	3 (27%)	0.4003
-ASCVD	5	2	
-Stroke	0	1	
-Heart failure	2	1	
-Hypertension	2	1	
History^b^ of kidney disease, *N* (%)	2 (17%)	2 (18%)	>0.9999
-Chronic kidney disease	0	2	
-End-stage renal disease	1	0	
-Tubular interstitial necrosis	1	0	

^a^T-Test.

^b^As listed in death certificate.

*N*, number of samples; SD, standard deviation; CVD, cardiovascular disease; ASCVD, atherosclerotic cardiovascular disease.

**Table 2:** Vascular calcification (VC), expressed as the percentage of calcified tissue relative to the total tissue volume [% by volume = (${\text{mm}}_{\text{calcified}}^{3}$/${\text{mm}}_{\text{total}}^{3}$)^*^100] in internal carotid arteries and brain regions analyzed by microCT.

	**Control, *N* samples**	**VC, median (25%−75% IQR)**	**Dementia, *N* samples**	**VC, median (25%−75% IQR)**	***p* (*T*-test, log-transformed)**
**Cohort 1**					
BG	12	0.0102 (0.0009, 0.2452)	11	0.0354 (0.0041, 0.4971)	0.2802
H	12	0.0023 (0.0002, 0.0072)	11	0.0231 (0.0156, 0.0772)	**0.0002**
PCC	12	0.0004 (0.0002, 0.0026)	11	0.0088 (0.0024, 0.0207)	**0.0272**
SvZ	11	0.00130 (0.0003, 0.0052)	9	0.0038 (0.0026, 0.0219)	0.3294
SN	9	0.0021 (0.0014, 0.0097)	11	0.0006 (0.0002, 0.0041)	0.2444
ICA	12	1.436 ± 1.726^a^	11	4.528 ± 4.120^a^	**0.0377** ^b^
**Cohort 1 (additional brain regions of interest)**					
Cb	6	0.0018 (0.0000, 0.0044)	8	0.0072 (0.0027, 0.0139)	**0.0484**
DPC	6	0.0000 (0.0000, 0.0010)	7	0.0012 (0.0003, 0.0020)	0.2609
LTC	6	0.0007 (0.0002, 0.0142)	7	0.0119 (0.0042, 0.0414)	0.0691
PG	6	0.0047 (0.0010, 0.0109)	8	0.0037 (0.0007, 0.0339)	0.7615
**Cohort 2 (NeuroBioBank control vs. late-onset AD)**					
PCC^a^	5	0.0024 (0.0016, 0.0104)	5	0.0153 (0.0075, 0.0581)	**0.0265**

^a^Mean ± SD.

Welch's *T*-test, non-transformed data.

SD, standard deviation; IQR, interquartile range; BG, basal ganglia; H, hippocampus; PCC, posterior cingulate cortex; SvZ, subventricular zone; SN, substantia nigra; ICA, internal carotid artery; Cb, cerebellum; DPC, dorsolateral prefrontal cortex; LTC, lateral temporal cortex; PG, parahippocampal gyrus.

Significant *p* values are highlighted in bold.

Based on interim analysis detecting significantly higher microvascular calcification in hippocampus and posterior cingulate cortex in dementia cases vs. controls, we expanded our study to: (1) sample additional brain regions from viable cadavers in cohort 1 [dorsolateral prefrontal cortex (BA 9), lateral temporal cortex (BA 21), parahippocampal gyrus (BA 36), and cerebellum were the additional regions of interest, based on publicly available transcriptomic data on differential gene expression between AD and age-matched controls in these brain regions (Greenwood et al., 2020); sample sizes obtained for each brain region are noted in Table 2], and (2) examine brain microvascular calcification in an additional cohort of subjects with clinically and neuropathologically confirmed late-onset AD (cohort 2).

Cohort 2 postmortem posterior cingulate cortex samples (BA 23, measuring ~1.5 cm^3^) were obtained (PMI < 24 h) from the Mt. Sinai Brain and Tissue Repository (a participating center in the NIH NeuroBioBank program) from human donors with (*n* = 5) and without (*n* = 5) clinically and neuropathologically diagnosed late-onset AD and no history of cardiovascular disease (CVD) who consented to allow their tissues to be used for medical research. The control samples provided matched the age and sex of the case samples provided (Table 3). The NIH NeuroBioBank is a shared resource, and as such, limits sample allocation per researcher. We were approved to conduct this pilot study on one brain region in ten total subjects. We chose to examine the posterior cingulate cortex, given our hypothesis that microvascular calcification may be the culprit underlying hypoperfusion in this region in subjects with AD (Small et al., 2000; Valla et al., 2001); moreover, while hippocampal calcification has been studied in AD (Tsolaki et al., 2022), calcification has not yet been examined in the posterior cingulate cortex. The Mt. Sinai Brain Bank operates in accordance with Bronx VA Medical Center Institutional Review Board (IRB) policies under IRB Protocol Number: HAR-13-059.

**Table 3:** Characteristics of the study population in Cohort 2 (NeuroBioBank late-onset AD cohort).

	**Control (*N* = 5)**	**AD (*N* = 5)**	***p*-value**
Sex, *N* female (%)	2 (40%)	2 (40%)	>0.9999^a^
Age, mean years ± SD	78 ± 2	78 ± 2	0.8910^b^
History of CVD, %	0	0	n.a.

^a^Fisher's exact.

^b^*T*-test.

*N*, number of samples; SD, standard deviation; CVD, cardiovascular disease.

Per the NIH definition of Human Subjects Research, the use of postmortem tissue is not considered Human Subjects Research and accordingly does not require IRB review of a research protocol by the requesting investigators. This study used only deidentified data and biospecimens, and the investigators did not have access to identifying information.

### 2.2 Micro-computed tomography (microCT)

Cohort 1 tissue samples were immersed in mineral oil in a plastic tube and scanned by micro-CT (Bruker SkyScan 1173) at 10 μm resolution (Figures 1A, B). Cohort 2 samples (embedded in paraffin blocks) were scanned at 15 μm resolution. Images were reconstructed and visualized in 3D (Micro Photonics NRecon and Object Research Systems Dragonfly software, Supplementary Video 1); standardized volumes of tissue (117.81 mm^3^ cylinders for brain tissue samples and 950.33 mm^3^ cylinders for internal carotid artery samples from cohort 1, and 1.2 cm^3^ cubes for brain tissue samples from cohort 2) were isolated as volumes of interest (VOI) and segmented at the intensity level equivalent to 130 Hounsfield units (HU), the threshold for detecting calcification in human tissue (Figure 1C) (Kopp et al., 2002). Vascular calcification volume (VC) was quantified as the bilateral sum of voxels ≥130 HU within each volume-standardized VOI (${\text{mm}}_{\text{calcified}}^{3}$/${\text{mm}}_{\text{total}}^{3}$). Internal carotid artery (ICA) VC measurements were taken immediately distal to the carotid bifurcation (extracranial C1-ICA segment). MicroCT findings were confirmed by histologic examination (Figure 1D). Histologic evaluation of the samples validated that the calcification detectable by microCT was specific to the vasculature; microCT was not sensitive to detect diffuse calcification (< 10 μm and/or < 130 HU) in the brain parenchyma. Calcification volumes < 0.002% were attributable to scattered hyperintensities captured during image segmentation. 3D-segmented microCT images were used to assess the prevalence of VC across brain regions sampled in cohort 1 cases and controls. Prevalence of VC was determined by the presence of at least one morphologically distinct microvessel showing any extent of segmented calcification (>130 HU) within a given brain region.

### 2.3 Histology

Non-decalcified tissue samples from cohort 1 were embedded in paraffin blocks, sectioned at 5 μm, and stained with calcium-binding alizarin red stain (pH 4.2). Paraffin-embedded tissue samples provided by the NeuroBioBank were also sectioned at 5 μm and stained with alizarin red. To examine calcification alongside other pathologies, serial sections from cohort 2 were stained with hematoxylin and eosin. All slides were imaged by bright-field microscopy using an Olympus BX53 microscope.

### 2.4 Statistical methods

**Figure 2:**
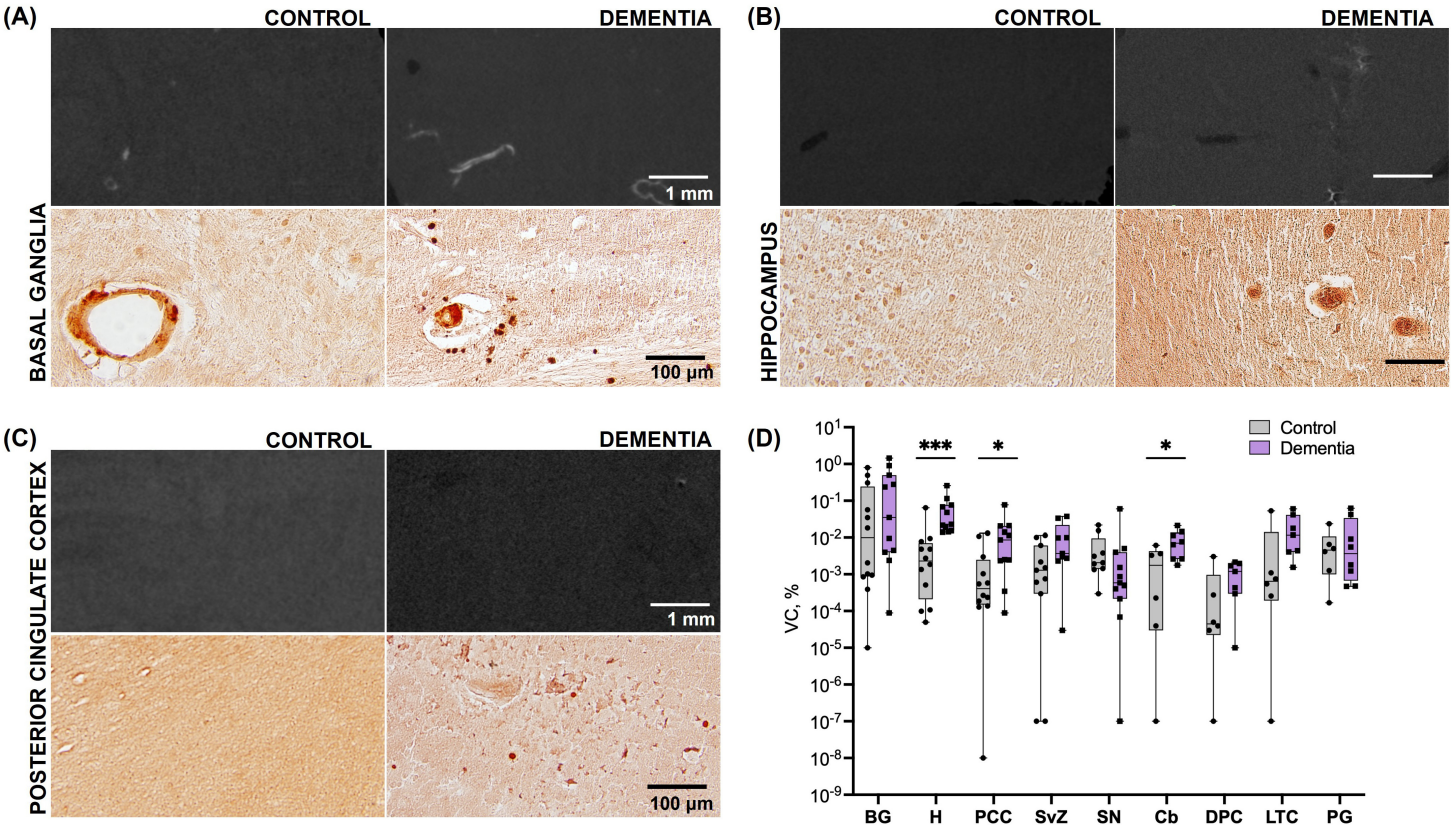
Calcification across brain regions in subjects with dementia vs. elderly controls (Cohort 1). **(A)** Representative microCT (top) and histologic (bottom) images of basal ganglia tissue in cadaveric subjects without dementia and with dementia. **(B)** Representative microCT (top) and histologic (bottom) images of hippocampal tissue in cadaveric subjects without dementia and with dementia. **(C)** Representative microCT (top) and histologic (bottom) images of posterior cingulate cortex tissue in cadaveric subjects without dementia and with dementia. **(D)** Vascular calcification, expressed as the percentage of calcified tissue relative to the total tissue volume of interest [(${\text{mm}}_{\text{calcified}}^{3}$/${\text{mm}}_{\text{total}}^{3}$)*100], across brain regions. Significant differences between case vs. control VC in H, PCC, and Cb were derived from pairwise comparisons in the log-transformed dataset; BG, basal ganglia; H, hippocampus; PCC, posterior cingulate cortex; SvZ, subventricular zone; SN, substantia nigra; Cb, cerebellum; DPC, dorsolateral prefrontal cortex; LTC, lateral temporal cortex; PG, parahippocampal gyrus. ^*^p < 0.05; ^***^p < 0.001.

**Figure 3:**
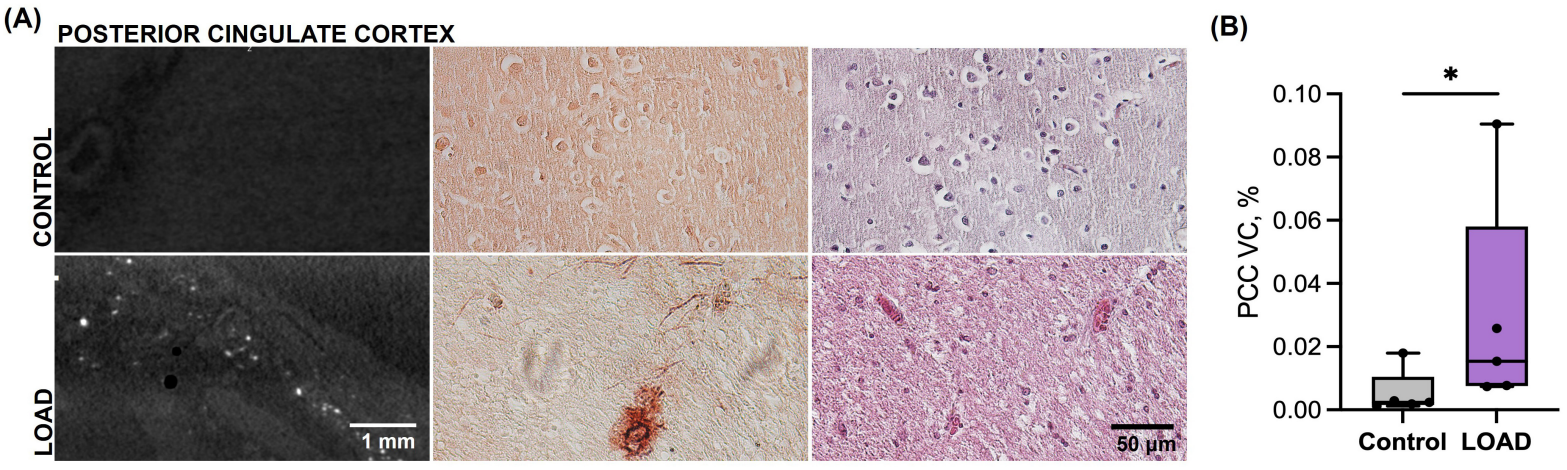
Posterior cingulate cortex calcification in subjects with late-onset Alzheimer's disease (LOAD) vs. elderly controls (Cohort 2). **(A)** Representative images from microCT (left), alizarin red staining (middle), and H&E staining (right) of posterior cingulate cortex tissue in control subjects and LOAD cases. **(B)** Comparison of PCC vascular calcification between LOAD cases vs. controls, expressed as the percentage of calcified tissue relative to the total tissue volume of interest ([${\text{mm}}_{\text{calcified}}^{3}$/${\text{mm}}_{\text{total}}^{3}$]*100). Error bars indicate the maximum and minimum values; box lines the upper quartile, median, and lower quartile. ^*^p < 0.05.

**Figure 4:**
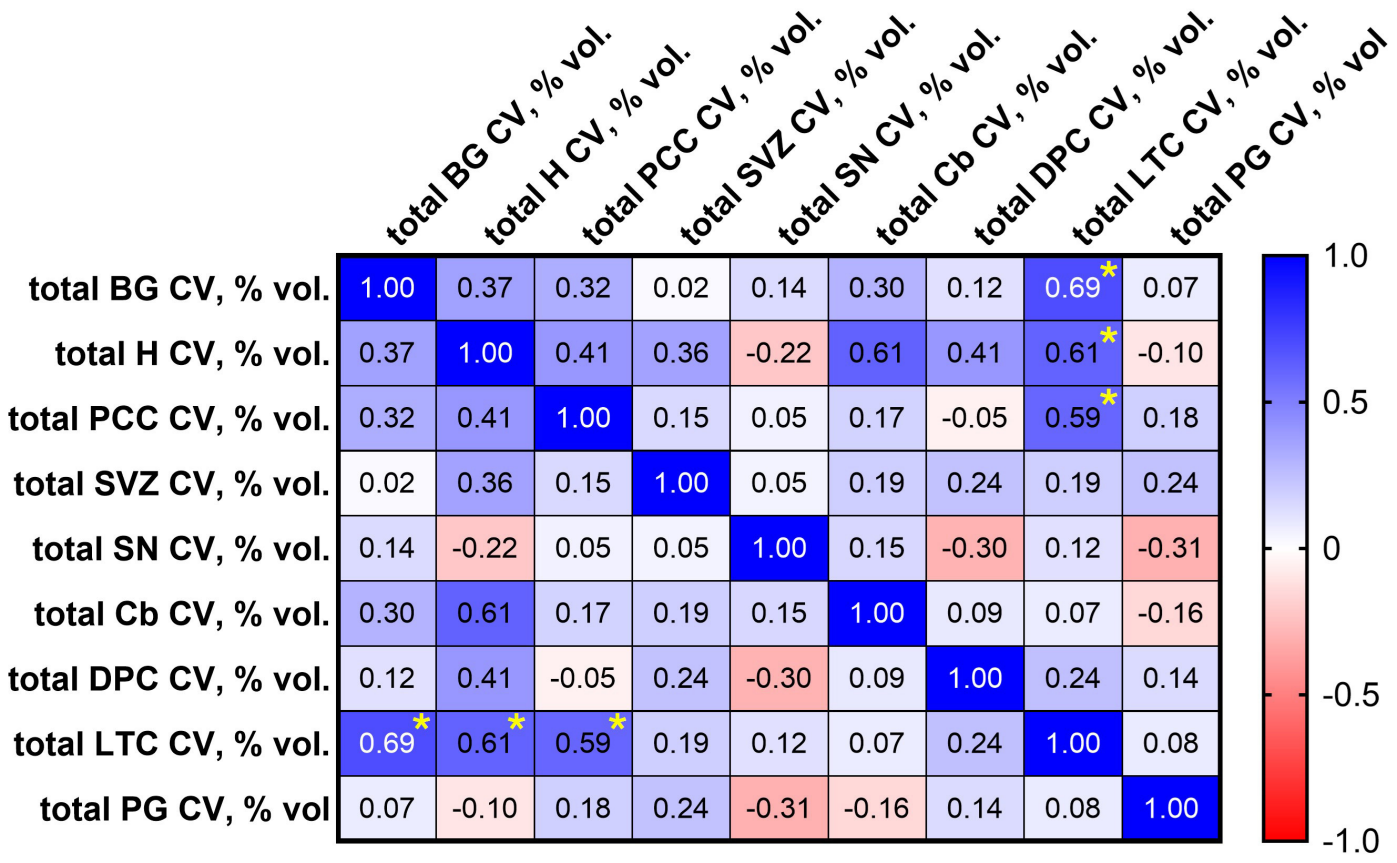
Spearman correlation matrix showing correlation between calcification volumes (% by volume = [${\text{mm}}_{\text{calcified}}^{3}$/${\text{mm}}_{\text{total}}^{3}$]*100) in brain regions sampled in cohort 1. Significant correlations, identified by asterisks (*), were observed between lateral temporal cortex (LTC) and basal ganglia (BG; *p* = 0.011), LTC and hippocampus (H) (*p* = 0.030), and LTC and posterior cingulate cortex (PCC; *p* = 0.036). SVZ, subventricular zone; SN, substantia nigra; Cb, cerebellum; DPC, dorsolateral prefrontal cortex; PG, parahippocampal gyrus.

Statistical analyses were conducted using GraphPad Prism version 10.2.3. Distributions of categorical variables were compared between groups by Fisher's exact tests (Tables 1, 2, Supplementary Table 1). Cohort 1 characteristics were obtained from the limited information listed on donors' death certificates; cohort 2 information was provided by the NeuroBioBank donor site. The normality of data was tested using the Shapiro-Wilk test. Brain microvascular calcification volumes, as measured by microCT (mm^3^), were expressed as the percentage of calcified tissue relative to the standardized total tissue volume [% by volume = (${\text{mm}}_{\text{calcified}}^{3}$/${\text{mm}}_{\text{total}}^{3}$)^*^100] in Table 2, Figures 2D, 3B, and Supplementary Table 2. Brain microvascular calcification volumes were not normally distributed (Supplementary Figure 1); as such, these were log-transformed. The association between left- and right- hemisphere calcification volumes in each brain region was tested by Pearson correlation analysis of log-transformed calcification (% by volume; Supplementary Table 2). Log-transformed calcification data (% by volume) were analyzed using unpaired Student's *T*-tests (Table 2, Figures 2D, 3B) to compare calcification between dementia cases and controls in each brain region. Internal carotid artery calcification (% by volume) data were parametric with unequal variance; as such, non-transformed data were analyzed by Welch's *T*-test to compare calcification between dementia cases and controls. Prior to running regression analyses, quartiles were defined for calcification volumes (mm^3^) in each brain region (Supplementary Table 3). Logistic regression analysis, using calcification volume quartile as a determinant and dementia as an outcome, was used to examine the odds of dementia per one quartile increase in calcification volume across brain regions sampled in cohort 1 (Table 4). Due to the small sample size, unadjusted regression estimates were reported. To examine the association between internal carotid artery calcification and brain microvascular calcification, linear regression analysis was performed using internal carotid calcification volume quartile as a determinant and brain microvascular calcification volume quartile as an outcome. Only adequately sampled brain regions (*n* ≥ 20) were included in these regression analyses. The correlation between calcification volumes in all sampled brain regions was further examined by Spearman correlation analysis on non-transformed data (Figure 4). No outliers were excluded in any of our analyses. Results from statistical analyses of parametric data are presented as mean ± standard deviation (SD), and from analyses of nonparametric data as median and 25%−75% interquartile range (IQR). *p* < 0.05 was accepted as statistically significant.

**Table 4:** Association between calcification volume (mm^3^, coded as quartile 1, 2, 3, or 4) and dementia.

**Vascular territory**	**Odds ratio (95% CI) per 1 quartile increase in calcification volume**	***p*-value**
ICA	1.923 (0.881, 4.825)	0.1211
BG	1.623 (0.758, 3.835)	0.2297
H	9.601 (2.518, 86.803)	**0.0091**
PCC	2.894 (1.222, 8.923)	**0.0302**
SVZ	2.851 (1.153, 9.482)	**0.0427**
SN	0.660 (0.270, 1.467)	0.322

CI, confidence interval; ICA, internal carotid artery; BG, basal ganglia; H, hippocampus; PCC, posterior cingulate cortex; SvZ, subventricular zone; SN, substantia nigra.

Significant *p* values are highlighted in bold.

## 3 Results

### 3.1 How does brain microvascular calcification compare between donors with vs. without dementia/late-onset AD?

Differences in calcification distribution, morphology, and volume were observed across brain regions and between the dementia cases and control groups in cohort 1. All dementia cases exhibited VC in the hippocampus (prevalence of VC by brain region is noted in Supplementary Table 1). MicroCT findings were confirmed by histologic examination, validating that the calcification detectable by microCT was specific to the vasculature (Figure 1D); microCT was not sensitive to detect diffuse calcification (< 10 μm and/or < 130 HU) in the brain parenchyma. Across all regions assessed, VC volumes, as measured by microCT, were highest in the basal ganglia in both cases and controls (Table 2). Basal ganglia calcification was dispersed throughout the brain parenchyma and vasculature in both cases and controls, as evident by microCT and histologic imaging (Figure 2A). Both vascular and parenchymal calcification were evident in the hippocampus and posterior cingulate cortex in dementia cases but not in controls (Figures 2B, C). Other sampled regions such as the dorsolateral prefrontal cortex and substantia nigra showed little to no calcification in both cases and controls (not depicted). VC was significantly greater in the hippocampus [case median 0.0231 (IQR 0.0156, 0772) vs. control median 0.0023 (IQR 0.0002, 0.0072), % by volume; *p* = 0.0002], posterior cingulate cortex [case median 0.0088 (IQR 0.0024, 0.0207) vs. control median 0.0004 (IQR 0.0002, 0.0026), % by volume; *p* = 0.0272], and cerebellum [case median 0.0072 (IQR 0.0027, 0.0139) vs. control median 0.0018 (IQR 0.0000, 0.0044), % by volume; *p* = 0.0484] in dementia cases compared to elderly controls without dementia; no significant differences between cases and controls were noted in other brain regions (Figure 2D, Table 2). In most regions, VC correlated between hemispheres (Supplementary Table 2).

Similarly, both vascular and parenchymal calcification was evident by histology in cohort 2 posterior cingulate cortex samples (Figure 3A). Significantly higher VC was measured by microCT in the AD cases compared to controls by microCT [case median 0.0153 (IQR 0.0075, 0.0581) vs. control median 0.0024 (IQR 0.0016, 0.0104), % by volume; *p* = 0.0265, Figure 3B].

According to logistic regression analysis on sufficiently sampled brain regions in cohort 1, odds of dementia were significantly higher per one quartile unit increase in calcification volume in the hippocampus [OR 9.601 (CI 2.518, 86.803), *p* = 0.0091], posterior cingulate cortex [OR 2.894 (CI 1.222, 8.923), *p* = 0.0302], and subventricular zone [OR 2.851 (CI 1.153, 9.482); Table 4].

### 3.2 How does extracranial internal carotid artery calcification compare between donors with vs. without dementia?

Although significantly greater internal carotid artery calcification was observed in dementia cases compared to controls (cases mean 4.528 ± 4.120 vs. control mean 1.436 ± 1.726, VC% by volume; *p* = 0.0377; Table 2), higher odds of dementia associated with increased internal carotid artery calcification did not reach statistical significance (Table 4).

### 3.3 How does calcification volume compare across vascular territories?

According to linear regression analysis, increased internal carotid artery calcification was significantly associated with increased microvascular calcification in the basal ganglia [OR 1.699 (CI 1.156, 2.496), *p* = 0.0093], hippocampus [OR 1.580 (CI 1.056, 2.366), *p* = 0.0281], and posterior cingulate cortex [OR 1.524 (CI 1.009, 2.299), *p* = 0.0452; Table 5]. By Spearman correlation analysis, significant correlations in microvascular calcification were identified between the lateral temporal cortex and basal ganglia [*r* = 0.692 (CI 0.2110, 0.9034), *p* = 0.011], lateral temporal cortex and hippocampus [*r* = 0.610 (CI 0.0705, 0.8733), *p* = 0.030], and lateral temporal cortex and posterior cingulate cortex [*r* = 0.593 (CI 0.0448, 0.8670), *p* = 0.036; Figure 4].

**Table 5:** Association between internal carotid artery (ICA) calcification volume and brain microvascular calcification volume (mm^3^, coded as quartile 1, 2, 3, or 4).

**Brain region**	**Odds ratio (95% CI) per 1 quartile increase in calcification volume**	***p*-value**
BG	1.699 (1.156, 2.496)	**0.0093**
H	1.580 (1.056, 2.366)	**0.0281**
PCC	1.524 (1.009, 2.299)	**0.0452**
SVZ	0.822 (0.495, 1.366)	0.4277
SN	0.835 (0.513, 1.360)	0.4472

CI, confidence interval; BG, basal ganglia; H, hippocampus; PCC, posterior cingulate cortex; SvZ, subventricular zone; SN, substantia nigra. Significant *p* values are highlighted in bold.

## 4 Discussion

### 4.1 Key findings of this study

To expand on the investigation of brain microvascular disease as a driver of dementia, our study utilized high-resolution microCT to quantify and compare internal carotid artery calcification and brain microvascular calcification in human donors with and without dementia and late-onset AD. Our findings of increased carotid and microvascular calcification volumes in dementia cases compared to controls align with observations of cerebral small vessel disease as a mediator between large artery disease and dementia (van den Beukel et al., 2023). Moreover, our cadaveric study findings were reproduced in an independent cohort (NeuroBioBank posterior cingulate cortex samples from subjects with and without late-onset AD). Our findings also support recent evidence of significantly greater brain tissue calcification in human donors with AD compared to elderly controls (*n* = 30; 38%, IQR: 35.33 vs. 4%, IQR: 9.515, *p* < 0.0001) (Tsolaki et al., 2022). The study by Tsolaki et al., however, did not distinguish calcification volumes specific to the vasculature, account for comorbid CVD among donors, or examine AD-relevant brain regions beyond the hippocampus and temporal cortex. All subjects in our cadaveric study exhibited VC in at least one brain region. Although our sample size was not large enough to estimate the prevalence of brain microvascular calcification in the elderly population, the prevalence in our sample exceeded published estimates of intracranial calcification across various elderly populations (Bartstra et al., 2020; de Brouwer et al., 2020; Yamada et al., 2013; Simoni et al., 2008). Interestingly, all dementia cases exhibited VC in the hippocampus, and 50% of the control group exhibited hippocampal VC. Further investigation is warranted to determine whether VC-related hypoperfusion of the hippocampus may contribute to age-related memory decline in the general population. Consistent with the literature, both the cases and controls sampled in our cadaveric cohort exhibited the greatest amount of VC in the basal ganglia relative to other brain regions studied. We cannot exclude the possibility that this may be due to a higher density of microvasculature in the basal ganglia relative to the other brain regions examined (Kubíková et al., 2018). Greater VC was observed in dementia (cohort 1) and late-onset AD (cohort 2) cases compared to elderly controls in brain regions relevant to morphologic changes (hippocampus) and hypoperfusion (posterior cingulate cortex) in AD. Our small study also found that the odds of dementia were higher with increasing calcification volume in both of these regions. The clinical significance of these findings is unclear, given (1) the lack of clinical imaging modalities for evaluating brain microvascular calcification and (2) the lack of a specific threshold for the volume of calcification considered pathological in the brain.

Clinical examination of the carotid arteries by ultrasonography is standard practice in assessing stroke risk, and carotid disease has been associated with dementia and cognitive decline (Bos et al., 2015; Geijselaers et al., 2016). A recent study of 272 adults found that carotid artery stiffness was associated with white matter hyperintensities and lacunes in the brain as well as impairment in global and domain-specific cognition (Robert et al., 2022). To isolate the relationship between carotid calcification (a major contributor to vascular stiffness) and brain microvascular calcification, we compared the calcification in a standardized volume of the extracranial internal carotid artery against microvascular calcification in a standardized volume in each brain region sampled. While we observed significantly greater carotid calcification in dementia cases compared to controls, higher odds of dementia associated with increased carotid calcification did not reach statistical significance; however, increased carotid calcification was significantly associated with increased microvascular calcification in brain regions relevant to AD/dementia. These findings align with the hypothesis that cerebral small vessel disease is a mediator between carotid disease and dementia (van den Beukel et al., 2023).

### 4.2 Vascular contributions to Alzheimer's disease and related dementias: Investigating the role of calcification

Like Aβ accumulation, VC is commonly encountered in advanced age; when extensively present in the young, it is usually attributable to a hereditary disorder such as primary familial brain calcification (PFBC) (Bartstra et al., 2020; Rutsch et al., 2020). The effects of VC on neurovascular function have not been examined beyond the context of PFBC, and the implication of VC in late-onset AD has not been established. Neurovascular calcification is the primary pathology involved in PFBC, a rare disease characterized by motor and/or cognitive deficits attributable to bilateral basal ganglia calcification. SPECT imaging studies demonstrating cerebral hypoperfusion in patients with PFBC suggest ischemic disruption of the basal ganglia-thalamocortical circuit due to extensive calcified vasculopathy as a plausible pathogenic mechanism of neurodegeneration and behavioral impairment in these patients (Chen et al., 2020). This observation is relevant in the context of our study because it offers precedent for studying brain microvascular calcification as a potential culprit of hypoperfusion in AD/dementia. The clinical significance of the calcification volumes measured in our study are unclear. Unlike the widespread clinical assessment of coronary artery calcification, a threshold level at which brain VC is regarded as pathological has not been defined.

A recent study on a mouse model of PFBC demonstrated that microglia regulate VC clearance via the triggering receptor expressed on myeloid cells 2 (Trem2) (Zarb et al., 2021). Studies in human tissue and in mouse models of familial AD demonstrate that the development of a specific microglial activation state responsive to Aβ-induced pathology, termed disease-associated microglia, is dependent on the expression of *TREM2* (Keren-Shaul et al., 2017; Ulland et al., 2017). The *TREM2*^*R*47*H*^ variant is associated with late-onset AD, and the mechanism of risk is likely in part by microglial dysfunction. Microglial dysfunction can present with intracranial VC and behavioral deficits, as observed in patients with mutations in genes implicated in microglial development (Bianchin and Snow, 2022; Nahar et al., 2019). Given the consequences of microglial dysfunction and its association with AD risk, future studies using models that incorporate AD-associated variants such as *TREM2*^*R*47*H*^ are necessary to determine whether intracranial VC occurs as a consequence of microglial dysfunction and contributes to AD pathophysiology by impairing local tissue perfusion. Additional studies on microglial gene expression in the setting of VC and other genetic and environmental risk factors for AD will further help to elucidate the role of VC in AD.

Intriguingly, transcriptomic data from over 1,100 human brain donors demonstrate that expression levels of genes associated with VC vary significantly across brain regions in donors with AD (Greenwood et al., 2020). For example, the expression of *ALPL* (the gene encoding the tissue-nonspecific alkaline phosphatase that promotes the formation of VC) is increased in the cerebellum and temporal cortex and decreased in the dorsolateral prefrontal cortex, while *TREM2* expression is increased in the dorsolateral prefrontal and posterior cingulate cortices and decreased in the cerebellum in donors with AD compared to age-matched controls. This observation suggests that differential gene expression may create conditions conducive to VC development (i.e., an increase in *ALPL* expression alongside a reduction in *TREM2* expression), potentially explaining some of the regional differences in VC, brain perfusion, and neurovascular pathology observed in AD. This gene expression hypothesis may explain our findings of increased cerebellar VC volumes in dementia cases compared to controls. Additionally consistent with this hypothesis, we did not observe VC in dorsolateral prefrontal cortex samples from dementia cases or controls.

### 4.3 Future directions

Additional studies are warranted to determine whether factors such as specific forms of CVD, risk factors for CVD, other comorbidities, or AD-associated gene variants potentially account for the increased VC observed in AD. *APOE4* is the strongest risk factor for both late-onset AD and cerebral amyloid angiopathy while also increasing the risk for atherosclerotic CVD and cerebrovascular disease (Mahley, 2016; Love and Miners, 2015). In mice, *APOE4* is associated with blood-brain barrier breakdown, decreased cerebral blood flow, and behavioral deficits independent of Aβ and phosphorylated tau (Montagne et al., 2021). Clarifying the vascular mechanisms by which LOAD-associated gene variants such as *APOE4* confer their risk will expand the discovery of therapeutic targets and biomarkers. Further, distinguishing whether increased neurovascular calcification in AD is merely associated with CVD or is an independent predictor of AD in a larger and more diverse study cohort is critical for elucidating its role in the pathophysiology of AD.

The vascular hypothesis of AD does not negate the Aβ hypothesis but suggests a more comprehensive disease mechanism (Scheffer et al., 2021). Vascular disease underlying hypoperfusion contributes to the hypothesis that AD arises as a consequence of mitochondrial dysfunction in these brain regions (Valla et al., 2001). Studying the role of VC as a driver of regional hypoperfusion and microglial dysfunction explores new concepts in the pathophysiology of AD. Establishing a mechanistic link between AD-associated gene variants, VC, and microglial dysfunction lends insight for therapeutic targeting of VC and microglia. Restoring arterial wall elasticity is the goal of VC-lowering therapy (Goettsch et al., 2020); this may improve tissue perfusion, perivascular drainage, and microgliosis in the brain.

### 4.4 Limitations of this study

Obtaining a sample size adequate for subanalyses by sex, CVD risk factors, and comorbidity status was not possible in the given study timeline. As such, we consider this a pilot study detecting a signal that links brain microvascular calcification and dementia; we hope our study will inspire a more comprehensive analysis. We acknowledge that our regression analyses imply each successive quartile increase in calcification is associated with the same increase in odds of dementia; we recognize that this assumption may not always hold; however, due to our limited sample size, modeling quartiles as categorical predictors or factors compared to a reference group results in relatively sparse data in each category and widened confidence intervals, limiting the reliability of the estimated odds ratios. Considering our small sample, our regression analyses allowed us to identify a general increasing trend between calcification and dementia. Our study is further limited by a lack of detailed medical history (i.e. death certificates were the sole sources of medical and demographic information available for cohort 1; medical information was not available for cohort 2 with the exception of the confirmed absence of CVD per our NeuroBioBank request), methodologic constraints of working with cadaveric tissue (i.e. availability of viable tissue samples, variability in fixation processes, and DNA degradation in embalmed tissue precluding genotyping ability) and limited availability of NeuroBioBank samples from brain regions of high interest in AD research, such that we were unable to obtain paraffin-embedded blocks of tissue from additional brain regions including the hippocampus (understandably, as this is a shared resource). The VC volumes reported in our study are expressed as a ratio over a standardized volume of brain tissue from each sampled region; this method of analysis does not account for the variation in vascular density across brain regions. Despite these limitations, our findings suggest that higher microvascular calcification in the hippocampus and posterior cingulate cortex is associated with dementia. Results from our regression analysis on the odds of dementia associated with increased calcification showed wide confidence intervals, likely due to our small sample size; as such, the odds of dementia per unit increase in calcification in the general elderly population is unclear. Future analyses on a larger sample, statistically adjusted for age, sex, and comorbidities, may offer a more accurate estimate of the relative risk of dementia associated with brain microvascular calcification.

## Data Availability

The raw data supporting the conclusions of this article will be made available by the authors, without undue reservation.
