# Supplementary material for: Brain microvascular calcification is increased in human donors with dementia compared to elderly controls: a pilot study
Source: Front Aging Neurosci. 2025 Jun 17;17:1557625. doi: 10.3389/fnagi.2025.1557625 (PMC12209190; doi:10.3389/fnagi.2025.1557625)
Supplement: Supplementary file 1 [file Supplementary_file_1.pdf]

Supplementary Material

1 Supplementary Tables

Supplementary Table 1. Prevalence of morphologically defined microvascular calcification by brain region (detected by microCT) in cohort 1

Region	Control		Dementia		P (Fisher's exact)
	N samples	Prevalence	N samples	Prevalence	
BG	12	58.3%	11	90.9%	0.1550
H	12	50.0%	11	100%	0.0137
PCC	12	25.0%	11	81.8%	0.0123
SvZ	11	36.4%	9	88.9%	0.0281
SN	9	33.3%	11	27.3%	>0.9999
Cb	6	50.0%	8	87.5%	0.2448
DPC	6	0%	7	0%	n/a
LTC	6	16.7%	7	85.7%	0.0291
PG	6	66.7%	8	50.0%	0.6270

BG, basal ganglia; H, hippocampus; PCC, posterior cingulate cortex; SvZ, subventricular zone; SN, substantia nigra; Cb, cerebellum; DPC, dorsolateral prefrontal cortex; LTC, lateral temporal cortex; PG, parahippocampal gyrus

Supplementary Table 2. Correlation between left- and right-hemisphere vascular calcification (% by volume = [$mm^3_{calcified}/mm^3_{total}$]*100); log-transformed) in each brain region.

Region	Pearson R	P
BG	0.630	0.002
H	0.535	0.015
PCC	0.622	0.004
SvZ	0.460	0.073
SN	0.248	0.338
Cb	0.729	0.003
DPC	0.625	0.040
LTC	0.113	0.756
PG	0.322	0.262

BG, basal ganglia; H, hippocampus; PCC, posterior cingulate cortex; SvZ, subventricular zone; SN, substantia nigra; Cb, cerebellum; DPC, dorsolateral prefrontal cortex; LTC, lateral temporal cortex; PG, parahippocampal gyrus

Supplementary Table 3. Calcification volume quartiles, as measured by microCT (mm^3)

	Q1	Median	Q3
ICA	7.763	26.18	117.7
BG	0.001227	0.02149	0.3628
H	0.002234	0.01679	0.05716
PCC	0.0002823	0.002831	0.01351
SvZ	0.0007768	0.003024	0.01067
SN	0.0005108	0.001792	0.004748
Cb	0.001661	0.004169	0.01030
DPC	3.980e-005	0.0003587	0.002215
LTC	0.0007789	0.004914	0.03500
PG	0.001263	0.005492	0.01523

Q, quartile; BG, basal ganglia; H, hippocampus; PCC, posterior cingulate cortex; SvZ, subventricular zone; SN, substantia nigra; Cb, cerebellum; DPC, dorsolateral prefrontal cortex; LTC, lateral temporal cortex; PG, parahippocampal gyrus

2 Supplementary Figure

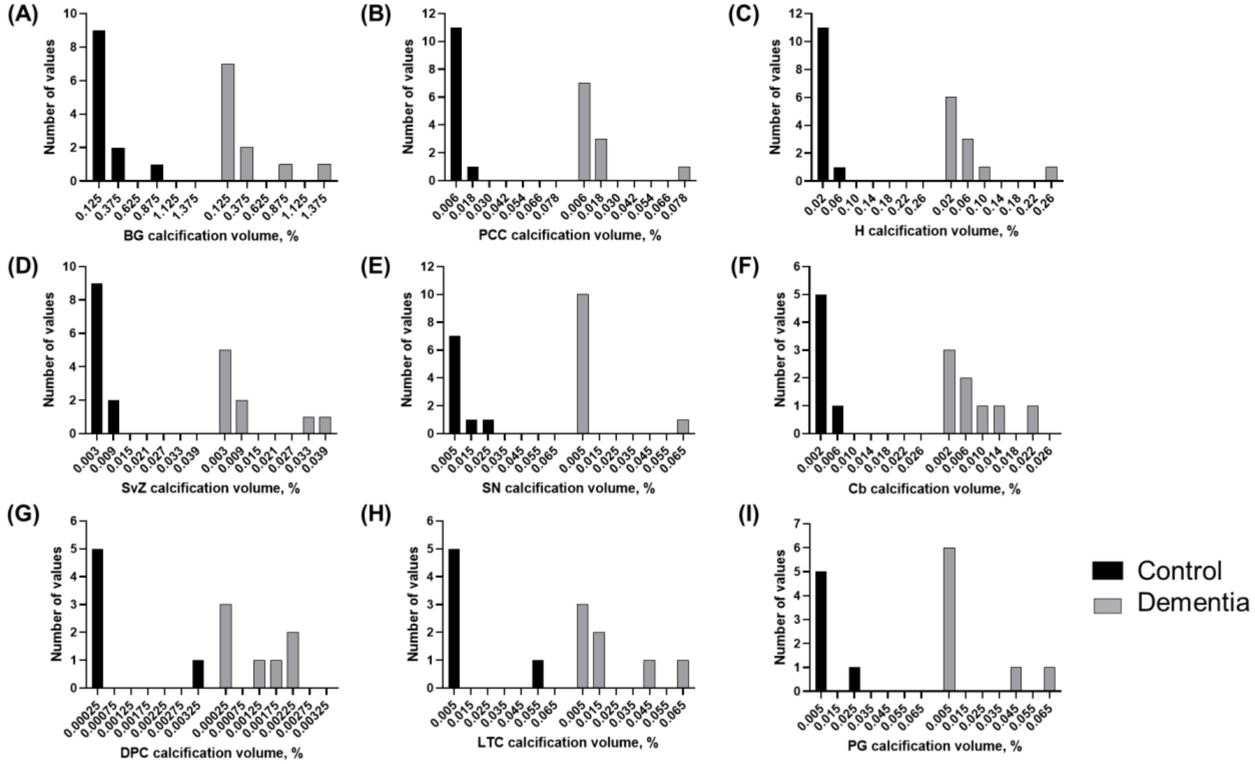

Supplementary Figure 1. Frequency distributions of vascular calcification volumes measured in various brain regions analyzed by microCT. (A.) Basal ganglia (BG), (B.) Posterior cingulate cortex (PCC), (C.) Hippocampus (H), (D.) Subventricular zone (SvZ), (E.) Substantia nigra (SN), (F.) Cerebellum (Cb), (G.) Dorsolateral prefrontal cortex (DPC), (H.) Lateral temporal cortex, (I.) Parahippocampal gyrus (PG); Note that calcification volumes of 0% are grouped into the smallest bin in each region. Tissue samples with calcification volumes $\leq$0.002% did not contain visible vascular calcification by microCT.

3 Supplementary Video

Supplementary Video. MicroCT 3D visualization using Dragonfly software (Object Research Systems). Vascular calcification visualized in the Y plane (top video) as the user scrolls through the X plane (bottom video).
